# Congenital Hepatic Arteriovenous Malformation Presenting as Isolated Massive Hepatomegaly in an Otherwise Healthy Neonate: A Case Report

**DOI:** 10.34763/jmotherandchild.2020241.2002.000008

**Published:** 2020-07-29

**Authors:** Charu Tiwari, Nilesh Nagdeve, Rajendra Saoji, Ghanshyam Hatwar, Sanskriti Sinha, Sharvil Thatte

**Affiliations:** 1 Department of Paediatric Surgery, GMCH, Nagpur, Maharashtra 440003, India; 2 Department of General Surgery, GMCH, Nagpur, Maharashtra 440003, India

**Keywords:** neonate, hepatomegaly, massive, arteriovenous malformation, hepatic

## Abstract

Congenital hepatic arteriovenous malformations (HAVMs), though rare, carry high morbidity and mortality rates if left undiagnosed. The usual clinical presentation is in infancy with congestive heart failure, anaemia and hepatomegaly. There are reports of presentation as persistent pulmonary hypertension in newborns and reports of their spontaneous regression as well. We describe a healthy full-term neonate with HAVM who was presented with isolated massive hepatomegaly and underwent surgical ligation.

## Introduction

Congenital hepatic arteriovenous malformation (HAVM) is a rare vascular anomaly characterised by direct fistulous arterial connection to a venous drainage system within the liver. The condition is characterised by a clinical triad manifesting clinically in neonates with congestive heart failure (CHF), anaemia and hepatomegaly (1, 2). Prenatal diagnosis is also possible when ultrasonography (USG) detects multiple engorged vascular channels in the foetal liver. The condition may be a cause of foetal loss due to high-output cardiac failure and hydrops. Morbidity and mortality rates are high if the condition is not recognised and treated promptly (3,4,5). Current treatment options include postnatal obliteration of the arterial feeder vessels by surgical ligation or percutaneous transcatheter embolisation with detachable coils (4).

In the present article, we describe a neonate with HAVM who was presented with massive hepatomegaly and was managed by hepatic artery ligation.

## Case Summary

**Figure 1:**
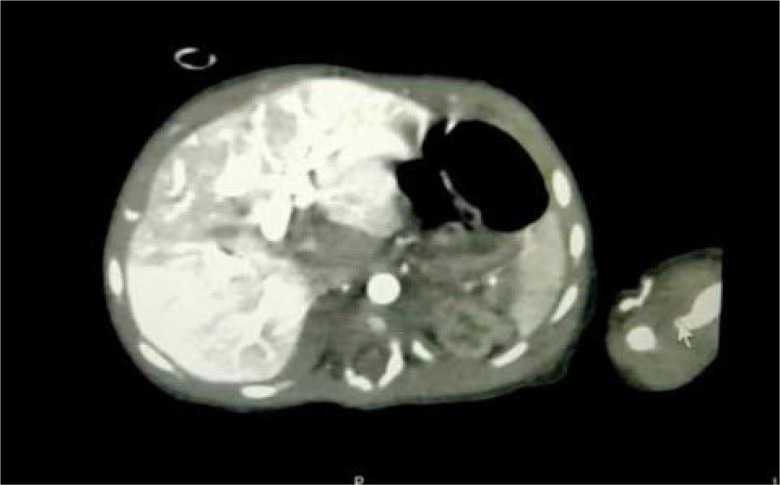
Contrast-enhanced CT scan showing early peripheral enhancement with central progression and central necrotic areas.

**Figure 2:**
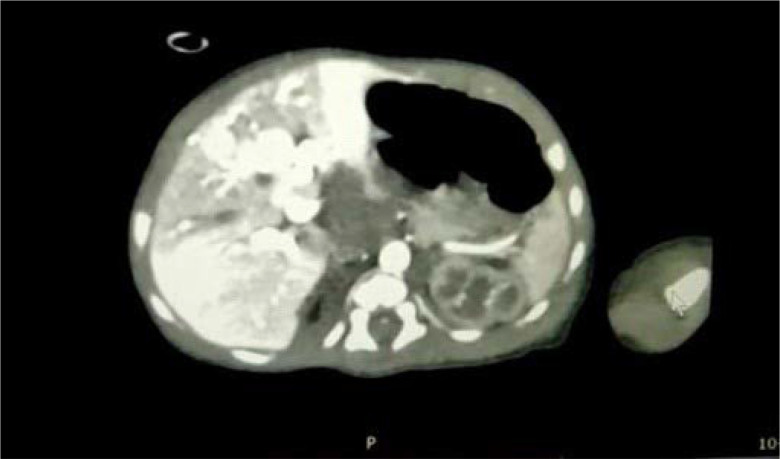
Contrast-enhanced CT scan showing mild enhancement or attenuation similar to that of adjacent parenchyma on delayed images and dilated celiac trunk.

**Figure 3:**
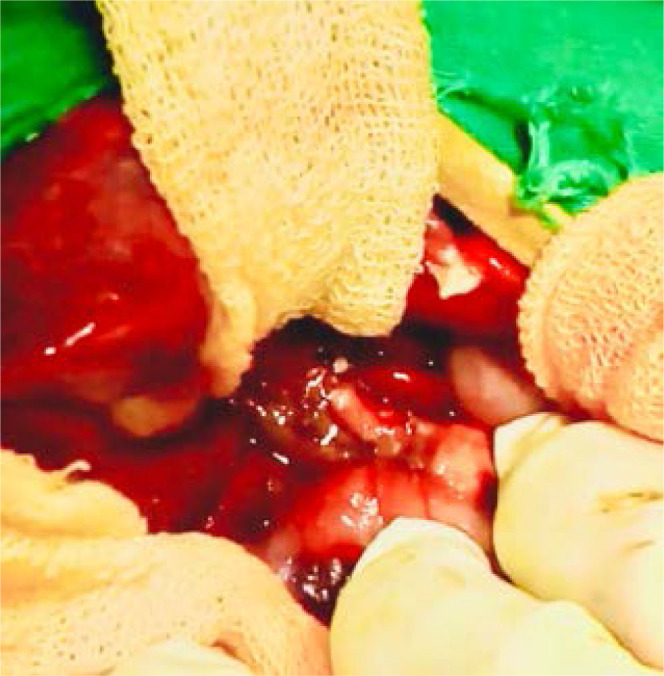
Intra-operative image showing the communication between the left hepatic artery and portal vein.

A 4-day full-term 3.5-kg male neonate referred to us for hepatomegaly. Antenatal details were not available. At admission, the baby was vitally stable; however, had early fatigue while taking feeds. He had an increased respiratory rate (56/min), but there were no subcostal/intercostal retractions. Hepatomegaly (12 cm below the right costal margin in mid-clavicular line) was presented and bruit could be felt over the liver and continuous machinery murmur could be auscultated. His haematological investigations, including platelet counts, were normal. Liver functions were also normal except for raised alkaline phosphatase levels. Abdominal USG revealed hepatomegaly with an anechoic cystic lesion of size 28 mm × 11 mm × 24 mm with surrounding dilated vessels noted in segments II and IVa of the left lobe of the liver with extension into segments V and VIII of the right lobe of the liver. Doppler US showed high-peak Doppler shifts and a low resistive index in the feeding arteries, hyper-pulsatility of the portal vein and a pulsatile pattern in draining veins. Unenhanced computed tomography (CT) scan demonstrated hepatomegaly and well-defined low-attenuation mass in the left lobe of the liver. Contrast-enhanced CT scans showed early peripheral enhancement with central progression, central necrotic areas and mild enhancement or attenuation similar to that of adjacent parenchyma on delayed images and dilated celiac trunk (Figures 1 and 2). Cardiac echo study was normal. Intervention radiologists were consulted for embolisation who refused intervention due to inexperience in doing such procedure in neonates. Hence, laparotomy and ligation of the left hepatic artery were done (Figure 3). The procedure and the postoperative period were uneventful. The patient was discharged on the eighth day.

On follow-up at 1 year, the baby was asymptomatic with normal liver function test. His hepatic size was grossly reduced. There was no recanalisation of HAVM on Doppler study.

## Discussion

The case reports of congenital HAVM are scarce, as a consequence of the rarity of the malformation itself. This condition has a 50–90% mortality rate, which emphasises the importance of a high degree of suspicion in achieving early diagnosis and definitive treatment (2, 5, 6). The most common clinical presentation includes CHF (58%) at an early age (mean 2.2 months) (7). Other presenting features include hepatomegaly, consumptive coagulopathy, anaemia, portal hypertension and hydrops fetalis (5). The combination of a brisk pulse, heart failure and absence of any major cardiac lesion or echocardiographic finding of significantly accelerated blood flow within the superior or inferior vena cava in the presence of a structurally normal heart should thus alert one to look for an AVM in a neonate (4, 8). There are isolated reports of HAVMs presented with persistent pulmonary hypertension. (1, 2, 7, 9). Our case was unique as though the HAVM has resulted in massive hepatomegaly, there were no other systemic complications such as congestive cardiac failure, thrombocytopenia and so on.

The etiopathogenesis and natural history of this condition are not well understood. HAVMs are known to be associated with autosomal dominant syndrome such as – Osler–Weber–Rendu syndrome, wherein angiodysplastic lesions are seen in the brain, lungs, liver, skin and mucous membranes. (10). Anatomically, HAVMs are simply direct arterial-to-venous communications that bypass the normal tissue perfusion capillaries. Multiple arterial feeder vessels draining into the AVM are also common (11). As the child grows, there is progressive dilation of the venous drainage owing to higher systemic blood pressure on arterial side, which results in characteristic sonographic findings of echogenic dilated vascular channels within the liver (11). More blood is shunted through this low-resistance, high-flow outlet and results in high-output heart failure, hydrops and even microangiopathic haemolytic anaemia, thrombocytopenia and consumptive coagulopathy known as the Kasabach–Merritt sequence (11). USG and colour Doppler study demonstrate dilated vascular channels in the parenchyma accompanied by enlarged hepatic arteries and veins (10). Venous spectral Doppler analysis shows arterialisation of venous waveforms (10). HAVMs appear as a tangle of dysplastic arteries and veins showing rapid enhancement on post-contrast imaging suggesting high blood flow, decreased resistive index on Doppler studies (10). Also, there is a lack of an associated soft tissue mass (which is seen in infantile hepatic haemangioma) (10). There is the presence of a nidus: the region of abnormal arteriovenous communication that bypasses the normal tissue capillary bed (10). Contrast-enhanced CT and MRI show confluent abnormal blood vessels with brisk enhancement. Spin echo MR sequences will typically demonstrate vascular flow voids secondary to the high flow nature of these lesions (10). Angiography acts as a tool to provide precise anatomical information as well as a means for endovascular therapy (1). Transarterial coil embolisation of critically ill neonates with AVMs enables the closure of the largest, highest flowing arteriovenous fistula, using umbilical artery access (12). However, the mortality rate of patients treated this way varies from 9 to 55% as reported in the literature (1).

Occlusion of the arterial feeder vessels by surgical ligation or percutaneous transcatheter embolisation is the optimum way of management (8, 11). However, there are isolated reports of HAVMs who improved or even regressed on medical therapy (3, 13). The mechanism attributed to spontaneous thrombosis of aneurysmal malformation, obstruction to the venous outflow tract and obstruction of the feeding artery (14). Transcatheter embolisation occludes the AVM nidus which is done via transarterial or transvenous (retrograde) approaches (11). It can be done in pre-term, low birth weight babies who are unstable and have single feeder artery. However, recanalisation has been reported leading to worsening of congestive cardiac failure (10). Surgical management options are ligation of feeding vessels, resection and liver transplantation. Medical therapy is directed at managing high output cardiac failure, pulmonary hypertension and consumptive coagulopathy. The mortality is due to congestive cardiac failure and persistent pulmonary hypertension. The reported incidence of mortality ranges between 50 and 75% (2, 5). Overall, the prognosis of an isolated hepatic AVM presenting in infancy is poor, especially in large lesions with multiple feeder vessels (1, 3, 6). Our patient was doing well until his first birthday.

In conclusion, we report a term neonate with HAVM presenting with massive hepatomegaly managed successfully by surgical ligation of the left hepatic artery.
